# Comparison of the Serum Concentration of Zinc in Patients With Bronchiectasis and Control Group

**DOI:** 10.5812/ircmj.7735

**Published:** 2013-07-05

**Authors:** Seyed Ali JavadMoosavi, Shahab Shahabi Shahmiri, Elyas Mostafapour, Mohammad Purfakharan, Mehran Zamanzadeh, Seyed Mohammad Fereshtehnejad, Hanieh Raji

**Affiliations:** 1 Tehran University of Medical Sciences, Tehran, IR Iran; 2 Tehran University of Medical Sciences and Health Services, Tehran, IR Iran; 3 Jundishapur University of Medical Sciences, Ahvaz, IR Iran

**Keywords:** Bronchiectasis, Zinc, Immune System

## Abstract

**Background:**

Bronchiectasis is an abnormal and permanent dilatation of bronchi. Infection plays a major role in causing and perpetuating bronchiectasis, as reducing the microbial load and attendant mediators are cornerstone of therapy. Zinc, as an integral micronutrient is involved in the immune reactions including response to infection. In several previous studies, mild zinc deficiency has been described in many infectious diseases such as abscess, cellulitis, chronic diarrhea, pneumonia, tuberculosis (TB), etc.

**Objectives:**

The purpose of this study was to determine serum zinc level in a series of patients suffering from bronchiectasis and to compare it with healthy control group.

**Patients and Materials:**

This analytical cross-sectional study was performed on thirty four patients with proven bronchiectasis and twenty nine healthy control subjects referred to Rasoul-e-Akram Hospital, Tehran, Iran, between March 2005 and March 2007. Serum concentration of the zinc was measured for all of the subjects and other information was completed according to their medical records. Both groups (case and control) were frequently matched regarding their age groups.

**Results:**

Patients included 11 (32.4%) males and 23 (67.6%) females with the average age of 55.03 (SD = 17.06) yr. The mean level of serum zinc in the case and control groups were 94.06 (SD = 20/96) mcg/dl and 103.7 (SD = 11.96) mcg/dl, respectively. Independent T-test analysis showed that serum zinc concentration in the case group of bronchiectasis patients was significantly lower than control group (P = 0.02).

**Conclusions:**

The results of our study show that serum zinc level in bronchiectasis patients was lower than the control group and the difference was statistically significant. It seems that the use of zinc supplement can reduce progression of the infectious disease regarding its role in improving the immune system reactions and some unknown mechanisms. Therefore, prophylactic and therapeutic use of zinc must be evaluated in further trials.

## 1. Background

Bronchiectasis is a syndrome of chronic cough and daily viscid sputum production associated with airway dilatation and bronchial wall thickening. Multiple conditions are associated with the development of bronchiectasis, but all require an infectious insult plus impairment of drainage, airway obstruction, and/or a defect in host defense. Treatment of bronchiectasis is aimed at controlling infection and improving bronchial hygiene (1, 2). Infection plays a major role in causing and perpetuating bronchiectasis, so that reducing the microbial load and attendant mediators are cornerstone of therapy (3). Bronchiectasis is a consequence of inflammation and destruction of the structural components of the bronchial wall. The host inflammatory responses induce epithelial injury, largely as a result of mediators released from neutrophils. As protection against infection is compromised, the dilated airways become more susceptible to colonization and growth of bacteria. On the other hand, zinc as an integral micronutrient, is involved in the immune reactions including response to infection (4, 5). Zinc deficiency is associated with impaired phagocytic function, lymphocyte depletion, decreased immunoglobulin production, a reduction in the T4+/T8+ ratio, and decreased interleukin (IL)-2 production (6-8). Zinc deficiency is prevalent in many developing countries and usually coexists with other micronutrient deficiencies (especially iron). Mild zinc deficiency has been described in many infectious diseases such as abscess, cellulitis, chronic diarrhea, pneumonia, tuberculosis (TB), etc. in several previous studies (9, 10). Whereas, zinc deficiency is not still completely evaluated in bronchiectasis patients and even a previous study has shown that serum zinc was in the same range of normal control groups (11).

## 2. Objectives

This study was conducted to determine serum zinc level in a series of patients suffering from bronchiectasis and to compare it with the value in control group.

## 3. Methods and Materials

### 3.1. Patients

This analytical cross-sectional study was performed on thirty four patients with proven bronchiectasis and twenty nine healthy control subjects referred to Rasoul-e-Akram Hospital, Tehran, Iran, between March 2005 and March 2007. The diagnosis of bronchiectasis was suspected when the patient had chronic daily cough with viscid sputum production and documented radiographically by the presence of bronchial wall thickening and luminal dilatation on high resolution chest computed tomographic (HRCT) scans (12). Serum concentration of the zinc was measured for all of the subjects and other information (such as radiologic evaluation of the site of pulmonary involvement, pulmonary function test (PFT), history of smoking and type of microorganism) was completed according to their medical records. Both groups (case and control) were frequently matched regarding their age groups.

### 3.2. Measurement

A 2 ml intravenous blood sample was taken from all subjects, and then taken in the heparinized and sterile test tube in -40° centigrade. Blood samples were withdrawn by zinc-free plastic syringes and placed in zinc-free centrifuge tubes. All samples were sent to Cellular and Molecular Research Center (affiliated with Tehran University of Medical Sciences & Health Services) for measuring serum concentration of zinc.

### 3.3. Statistical Analysis

The data were analyzed using SPSS v.16 software for Windows (SPSS Inc, Chicago, IL, USA). Parameters such as frequency, mean, mode and standard deviation (S.D.) were reported. The analyses were performed using statistical tests. Kolmogorov Smirnov (K.S) test was performed to evaluate normal distribution of the quantitative variables. All of the variables were compared using independent T test (for quantitative variables) and the chi-square test (for qualitative variables). In addition, correlation analysis was used to evaluate the association between different continuous variables of the study. A 5% probability of a type I error (two-tailed), and a power of 80% were considered in the analysis. All reported p-values are two-tailed.

## 4. Results

The case group of bronchiectasis patients included 11 (32.4%) males and 23 (67.6%) females with the average age of 55.03 (SD = 17.06) year and the control group of healthy individuals included 17 (58.62%) males and 12 (41.38%) females with the average age of 57.86 (SD = 14.16) yr. Serum zinc level of both patients and control group were measured. The mean serum zinc level in case and control groups were 94.06 (SD = 20.96) mcg/dl and 103.7 (SD = 11.96) mcg/dl, respectively. The results of Independent T-test analysis showed that serum zinc concentration in case group was significantly lower than the control group (P = 0.02). More detailed analysis was performed regarding the subjects’ gender. It was shown that there is a significant difference in serum zinc level of bronchiectasis patients and healthy controls in female gender (P = 0.03); whereas, this difference was not statistically significant between the male gender of the two groups (P = 0.3). Moreover, there was not any significant association between subjects’ age and the serum concentration of zinc (P = 0.49).

## 5. Discussion

The results of our study show that serum zinc level in patients suffering from bronchiectasis was significantly lower than the control group. To date, several studies have evaluated the role of serum zinc level in a series of patients with infectious diseases such as pneumonia, diarrhea and acute respiratory infection but only a few studies have evaluated serum zinc level in bronchiectasis patients (11), which have different and controversial results (9-11, 13). Therefore, it makes our results more considerable. In 2007, Meydani et al. (9) observed that elderly nursing home residents with low serum zinc had a higher risk of pneumonia, a longer duration of pneumonia episodes, a greater number of new antibiotic prescriptions, and more days of antibiotic use for the treatment of pneumonia. The results of another study by Faghihinia et al. (10) in 2001 indicated that zinc deficiency and pneumonia were significantly related. On the other hand, in one of the few similar studies on bronchiectasis patients, the mean serum zinc levels and the range of individual values in Beeley’s study of 65 patients with bronchiectasis have been shown to be of the same order as those of two separate groups of normal control subjects (11). Moreover, the study by Chang et al (13) in 2006 did not support the use of vitamin A or zinc supplementation in the management of acute lower respiratory infection requiring hospitalization in indigenous children (13).

Zinc, as a micronutrient, plays an important role in the regulation of the T cell–mediated function (14-16); Even though, zinc deficiency has been shown to cause thymus involution and to depress lymphocyte proliferation, interleukin-2 (IL-2) production, delayed-type hypersensitivity skin responses, and antibody response to T cell–dependent antigens (14, 17). Furthermore, zinc supplementation has been shown to improve T cell–mediated function in the elderly (18-22). In children, low concentrations of circulating zinc is associated with an increased risk of respiratory morbidity (23) and in some studies, zinc supplementation has been shown to reduce both the risk and duration of pneumonia and deaths due to pneumonia in children. Zinc (20) (mg/d) may be an effective adjunctive therapeutic strategy for diarrheal disease in children (24). It seems that the use of zinc supplement can reduce progression of the infectious disease regarding its role in improving the immune system reactions and some unknown mechanisms. Our study has some limitations regarding cross-sectional designing, not evaluating the longitudinal changes of serum level of zinc and the small sample size. However, it is one of the first studies to show the importance of zinc deficiency in patients suffering from bronchiectasis. Therefore, it is suggested to evaluate the prophylactic and therapeutic use of zinc supplementation in further trials and/or longitudinal assessments of bronchiectasis patients.
